# *Pneumocystis jirovecii* dihydropteroate synthase gene mutations in a group of HIV-negative immunocompromised patients with *Pneumocystis* pneumonia

**DOI:** 10.3892/etm.2014.2002

**Published:** 2014-10-03

**Authors:** YINGJIAO LONG, CHENG ZHANG, LI SU, CHENGLI QUE

**Affiliations:** Department of Pulmonary Medicine, First Hospital, Peking University, Beijing 100034, P.R. China

**Keywords:** *Pneumocystis jirovecii*, drug resistance, dihydropteroate synthase gene, mutation

## Abstract

The purpose of this study was to investigate dihydropteroate synthase (DHPS) mutations and their clinical context in non-HIV-infected patients with *Pneumocystis* pneumonia (PCP). DHPS genes in respiratory samples collected from HIV-negative patients with PCP presented between January 2008 and April 2011 were amplified by polymerase chain reaction (PCR) and sequenced. Basic clinical data from the medical records of the patients were also reviewed. The most common point mutations, which result in Thr55Ala and Pro57Ser amino acid substitutions, were not detected in the *Pneumocystis jirovecii* sampled from the HIV-negative patients. Two other point mutations, which result in nonsynonymous mutation, Asp90Asn and Glu98Lys, were identified in *P. jirovecii* from two patients. Among the patients, the levels of lactate dehydrogenase (LDH), C-reactive protein (CRP) and plasma (1–3) β-D-glucan were elevated in 75, 92.31 and 42.86% of patients, respectively. The percentage of circulating lymphocytes was significantly lower in non-survivors than in survivors [4.2%, interquartile range (IQR) 2.4–5.85 versus 10.1%, IQR 5.65–23.4; P=0.019]. The neutrophil proportion in bronchoalveolar lavage fluid (BALF) was significantly higher in non-survivors than in survivors (49.78±27.67 versus 21.33±15.03%; P=0.047). Thirteen patients had received adjunctive corticosteroids (1 mg/kg/day prednisone equivalent) and nine (69.23%) of them eventually experienced treatment failure. No common DHPS gene mutations of *P. jirovecii* were detected in the HIV-negative PCP patients. However, other mutations did exist, the significance of which remains to be further identified. The elevation of neutrophil counts in BALF and reduction of the number of lymphocytes in peripheral blood may be associated with poor outcome. The efficacy of adjunctive steroid therapy in HIV-negative patients with *P. jirovecii* infection requires further investigation.

## Introduction

*Pneumocystis* pneumonia (PCP), which is caused by *Pneumocystis jirovecii* (formerly called *Pneumocystis carinii* f. sp. *hominis*), is among the most prevalent opportunistic infections among immunocompromised patients, especially in patients with AIDS. In the past, PCP was generally indolent with latent onset; however, in HIV-negative patients, it usually progresses rapidly to severe hypoxemia, or even respiratory failure (1) with high mortality.

Currently, co-trimoxazole, (a 1:5 mixture of trimethoprim and sulfamethoxazole; TMP-SMZ) remains the first-line and most effective regimen for anti-*Pneumocystis* therapy and has been widely used for prophylaxis in patients at high risk for PCP since the 1990s. The widespread application of co-trimoxazole has been implicated in the increases in sulfa drug-resistant bacteria reported in HIV-infected patients. It has also raised concerns about the possible selection of drug-resistant *Pneumocystis*. In addition, a number of studies have confirmed that mutations in the *Pneumocystis* dihydropteroate synthase (DHPS) gene, which encodes the target enzyme that is combined with and inhibited by sulfamethoxazole, confer the sulfa drug resistance. The most frequently reported mutations are at positions corresponding to codons 55 and 57, which result in amino acid changes (2,3), that is, nonsynonymous mutation. Furthermore, there are also reports of mutations on codons 23 (2), 111 (2), 248 (2), 171 (4) and 60 (5).

So far, genetic and epidemiological data concerning *P. jirovecii* infections in China are rather scarce, and mainly relate to isolates from HIV-infected patients. Kazanjian *et al* (6) revealed a DHPS gene mutation rate of 7% (1/15) in AIDS patients with PCP in Beijing. However, Li *et al* (7) did not find mutations in the DHPS gene in *P. jirovecii* in 10 HIV-infected patients in Guangzhou, China. To the best of our knowledge, no data have been reported concerning *Pneumocystis* DHPS gene mutations in HIV-negative patients in China.

The objective of the present study was to investigate the presence of DHPS gene mutations in *P. jirovecii* from non-HIV-positive patients with PCP from a major general hospital in China. Clinical information, including certain laboratory parameters, treatment and outcome were also reviewed, to determine the effect of mutation of the *Pneumocystis* DHPS gene on clinical outcome in HIV-negative patients.

## Methods

### Patients

In this retrospective study, 22 non-HIV-positive patients with PCP, which were confirmed by Gomori methenamine silver (GMS) staining of respiratory samples, were included. The patients attended Peking University First Hospital, a 1,368-bed teaching hospital in Beijing, China between January 2008 and April 2011. The present study was approved by the Institutional Review Board of Peking University and was performed in accordance with the recommendations of the Helsinki Declaration of 1975. Written informed consent was obtained from all patients.

### Materials

A total of 24 respiratory samples, comprising 22 bronchoalveolar lavage fluid (BALF) and two sputum samples, were obtained from the 22 HIV-negative patients with confirmed PCP. The patients’ medical records were also reviewed and the outcome was followed up.

### Sample processing and DNA extraction

All the samples were dissolved with dithiothreitol (DTT) first, and then filtered with a nylon mesh and centrifugation. Part of each sample was stained and demonstrated to be GMS positive, and the remainder was stored at −20°C. DNA extraction was performed using E.Z.N.A. Blood DNA kit (Omega Bio-Tek Inc., Norcross, GA, USA).

### Polymerase chain reaction (PCR)

PCR was performed to analyze the DHPS gene of *Pneumocystis*, using the previously reported primers DHPS-3 and DHPS-4 (8). The reaction mixture contained 2.5 μl DNA template, 12.5 μl Taq PCR Master Mix (Qiagen, Valencia, CA, USA), 1.5 μl (10 μM) forward primer, 1.5 μl (10 μM) reverse primer and sterile water, making a total volume of 25 μl. PCR was used to amplify the samples, yielding a 370-bp fragment. A hot-start step at 94°C for 5 min was followed by the following for 45 cycles: DNA denaturation at 94°C for 30 sec, annealing at 61°C for 30 sec and extension at 72°C for 30 sec. This was followed by a final extension step at 72°C for 5 min. The amplification products were analyzed by electrophoresis on a 1.5% agarose gel containing ethidium bromide, and the bands were visualized with ultraviolet light. To prevent contamination, all PCR procedures were performed with a negative control of sterile water.

### Sequencing

The PCR products were sent to a genomic company (SinoGenoMax Co., Ltd, Beijing, China) and sequenced using an automated DNA sequencer (ABI 3730xl; Applied Biosystems, Foster City, CA, USA). Sequence analysis was performed using BLAST (http://blast.ncbi.nlm.nih.gov/Blast.cgi) and Clustal W (http://www.ebi.ac.uk/Tools/msa/) software.

### Statistical analysis

Clinical information, including laboratory results, therapy and outcome of patients were collected from medical records. SPSS software, version 17.0 (SPSS, Inc., Chicago, IL, USA) was used to analyze data, and the Student’s t-test was used to assess significant differences in continuous data with Gaussian distribution, while the Mann-Whitney U-test was used for non-normal distribution. Proportions between groups were compared using the χ^2^ test. A P-value of <0.05 was considered to indicate a statistically significant result.

## Results

### Demographic and clinical characteristics

Twenty-two specimens from bronchoalveolar lavage and two sputum samples were obtained from 22 HIV-negative patients with confirmed PCP from January 2008 to April 2011. Twenty-one DHPS gene fragments (that include the most frequently reported mutations) from 20 patients were successfully extracted from 24 (87.5%) samples. Among these 20 patients, two patients were outpatients whose medical records were unable to be retrieved. The demographic characteristics of the other 18 patients and underlying diseases of these patients are listed in Table I. All patients had received immunosuppressive agents, but none of them had ever received prophylaxis against PCP. One patient (5.6%) had experienced a prior episode of PCP. Table II shows that lactate dehydrogenase (LDH) levels were above the upper limit of normal in 9/12 patients (75%). β-D-glucan levels were elevated in 10/14 patients (71.3%). The CD4^+^ lymphocyte count was <200/μl in 9/10 patients (90%).

The 28-day mortality rate was 50%. Table III shows the prognostic factors that were identified to be associated with mortality by univariate analysis. These were peripheral neutrophils (P=0.003), peripheral lymphocytes (P=0.019) and neutrophils in BALF (P=0.047). The treatment and related mortality were also analyzed and are summarized in Table IV. Caspofungin therapy was administered to eight patients (50%) and 75% of them failed to survive. Thirteen patients had received adjunctive corticosteroids (1 mg/kg/day prednisone equivalent) and nine (69.23%) of them succumbed.

### DHPS gene mutations

No mutations in the DHPS gene were detected in the sequenced amplicons at codons 55 or 57. All had the wild-type genotype with the nucleotide sequence ACA CGG CCT at codons 55, 56 and 57, respectively, corresponding to threonine and proline at positions 55 and 57.

However, gene mutations at two relatively rare positions were identified. One mutation was observed at DHPS codon 98 in two patients with PCP, with glutamate replaced by lysine. The other was at DHPS codon 90 in a sample obtained from one of these two patients, with aspartate replaced by asparagine. The patient with only one mutation at codon 98, a 51-year-old female with dermatomyositis, developed PCP during adjustment of the dose of corticosteroids and finally succumbed following active treatment with TMP-SMZ. The patient with two mutations was a 31-year-old male kidney transplant recipient. The manifestations of this patient were mild with a PaO_2_ of 83.29 mmHg on ambient air. The mycophenolate mofetil dosage of the patient was halved immediately following the diagnosis of PCP, while the FK506 dosage remained unchanged. This patient declined TMP-SMZ treatment and self-discharged. The patient was followed up by telephone and it was reported that his condition had improved.

## Discussion

The present study is, to the best of our knowledge, the first that has investigated DHPS gene mutations in HIV-negative PCP patients in China. The results of the present study revealed that the most common point mutations, which result in Thr55Ala and Pro57Ser amino acid substitutions, were not detected in the *P. jirovecii* isolates from any of the samples from 20 non-HIV patients with confirmed PCP. This result is consistent with that of previous studies in HIV-positive patients (6,7), and suggests that the prevalence of DHPS gene mutations remains low in China. A number of studies have found that the prevalence of DHPS gene mutations in developed countries is relatively higher than that in developing countries with the highest prevalence being 72% in the USA (6,9). It is hypothesized that the relatively lower prevalence in developing countries, for example, 6.2% in India (10) and 56% in Africa (11), may be due to the reduced use of sulfa prophylaxis. It has been reported that even short-term exposure to TMP-SMZ can be associated with the emergence of resistance (12). According to the literature, the major antipneumocystic activity of this agent for PCP is derived from sulfamethoxazole (13) and the trimethoprim component is a very poor inhibitor of *P. jirovecii* DHFR (dihydrofolate reductase) (14). A number of studies have documented an association between the failure of sulfa prophylaxis and the occurrence of mutations in the *P. jirovecii* gene coding for DHPS, especially at codons 55 and 57, either as single mutations or combined (15,16). One study reported that DHPS mutations were significantly more common in patients who had previous exposure to sulfa drugs (18/29; 62%) than in those who had no exposure (13/123, 10.5%; P<0 0001) (5). In another study, which included 158 patients of five hospitals in France, multivariate analysis of risk factors for DHPS gene mutation revealed that sulfa prophylaxis is among the independent risk factors (adjusted odds ratio, 26.04; P<0.001) (5).

It has been suggested that the geographic area of residence, which may reflect the resistant strains, is an independent predictor of the harboring of DHPS mutations (17). No common mutations of the DHPS gene were detected in the present study. This result may be associated with the characteristics of the population that was studied. Kazanjian *et al* (18) have also reported that the duration of prophylaxis increases the risk of mutations [relative risk (RR) for each exposure month, 1.06; P=0.02] and that there is a statistically significant increase in the presence of a DHPS mutation if the duration of sulfa exposure is >1 year (RR, 1.16; P=0.003); however, the dose of sulfa was not found to be significantly associated with the mutation. The present study focused on HIV-negative patients and none of them received any sulfa prophylaxis. Although some of the patients in the present study (10/18) received sulfa drug treatment prior to BALF sampling, this was of little consequence due to the short exposure time (<7 days).

Patients with mutant DHPS genotypes are more likely to have severe disease, require invasive ventilation and have a poor outcome than patients with wild-type DHPS genotypes (19,20). In the current study, 72.2% of patients had hypoxemia and 66.7% of patients received mechanical ventilation. Although TMP-SMZ was used as first-line treatment, with the exception of contraindication as soon as PCP was suspected, the 28-day mortality rate remained as high as 50%. It is assumed that DHPS gene mutation may be just one of the mechanisms of sulfa resistance. A number of other factors may also play a role, such as pharmacokinetics/pharmacodynamics (PK/PD), underlying diseases, nutritional status and complications. The timing of the first dose of sulfa administration is likely to be crucial to the outcome of patients. In addition, the full length of the DHPS gene was not examined to exclude the possibility of existence that other mutation positions exist that are responsible for sulfa-drug resistance, because a previous study has done so and found no such mutations (5). As certain patients harbouring *Pneumocystis* with DHPS gene mutations respond to treatment with high doses of TMP-SMX, a possible explanation is that high-dose therapy may compensate for reduced sensitivity (21). However, in the present study, all patients received 15 mg/kg of TMP, or an adjusted dose according to renal function.

With the absence of common mutations, two other nonsynonymous point mutations, Asp90Asn and Glu98Lys, were identified in the *P. jirovecii* isolates from two patients who had different underlying diseases and clinical manifestations, as well as completely different outcomes. The implications of these mutations call for larger scale study.

The immune backgrounds of the patients in the current study were similar to those in a previous study (9). However, none of the patients in the present study had ever received prophylaxis, despite the fact that in nine of 10 patients, the CD4^+^ lymphocyte count was <200/μl on admission. It has been suggested that prophylactic treatment should be applied to HIV-positive patients with CD4^+^ lymphocyte counts <200/μl (10). However, there is no such agreement for HIV-negative immunosuppressed patients. In clinical practices, physicians have to weigh benefits against risks to make an appropriate decision.

The onset symptoms of PCP were variable and nonspecific, including fever, cough, dyspnea and chest tightness. It has been suggested that the level of serum β-D-glucan and *S*-adenosylmethionine is diagnostic for PCP within the appropriate clinical context (22) and the level of LDH is elevated at an early stage, offering diagnostic value despite its low specificity (23). In the present study, 75% of patients had elevated LDH levels and 92.31% of them had elevated CRP levels, supporting the high sensitivity of these indicators. In addition, 71.43% of patients had elevated β-D-glucan levels, which is lower than the sensitivity of 98% and specificity of 94% reported by a previous study (24). The results of the present study showed that serum β-D-glucan levels were markedly elevated in 42.86% of patients; however, the reduction of the level with therapy did not translate into survival. At present, the gold standard to confirm PCP remains the microscopic examination of respiratory samples. However a low fungal load in HIV-negative patients with PCP (25) may lead to false negativity (26). Several PCR assays have been developed with higher sensitivity and specificity, but the detection of *Pneumocystis* DNA provides no information concerning the organism’s viability or infectivity, and therefore cannot exclude the possibility of colonization in asymptomatic patients (27), particularly in patients receiving corticosteroid therapy or immunocompetent patients with lung disease (28,29). In the present study, 55.6% of the patients received sulfa pre-emptive treatment prior to bronchoalveolar lavage, which may have exerted an influence on the positive rate of microscopy. The results showed that the lymphocyte count was significantly lower in nonsurvivors than in survivors (4.2%, IQR 2.4–5.85 versus 10.1%, IQR5.65–23.4; P<0.05), which supports the theory that cellular immunity plays an important role in anti-pneumocystic therapy, particularly that involving CD4^+^ T cells. Similarly, the CD4^+^ T cell count in nonsurvivors was lower than that in survivors, although the difference was not significant statistically due to missing data. In univariate analysis, it was found that the neutrophil count in the BALF from nonsurvivors was significantly higher than that from survivors (49.78±27.67 versus 21.33±15.03%; P<0.05), indicating the possibility that the elevation of the neutrophil count in BALF may be associated with a poor prognosis. The limited number of samples hampered the multivariate analysis.

In this study, 16 out of 18 patients were treated with co-trimoxazole as a first-line therapy; only five patients were treated with the intravenous form. According to the literature, the bioavailability via oral or intravenous administration is thought to be equivalent (30). Caspofungin, an inhibitor of fungal 1,3-β-D-glucan synthesis (31,32), is effective for the treatment of invasive candidiasis and aspergillosis (33). There are some case reports indicating its effectiveness as a salvage or additional treatment for PCP (34,35). Caspofungin has strong activity on cyst forms and weak activity on trophic forms, whereas TMP-SMZ mainly interferes with trophic forms. Theoretically, the concomitant use of TMP-SMZ and caspofungin, by fully inhibiting the organism life cycle, may provide synergistic activity against *Pneumocysitis* (36). In the present study, eight patients (50%) received caspofungin therapy and 75% of them failed to survive. However, caspofungin is usually recommended in severe cases, and this may have affected the mortality rate. In addition, the number of patients was also limited in the present study. Adjunctive corticosteroids, in addition to antibiotics, are of substantial benefit in HIV-infected patients with moderate to severe hypoxemia (37,38). In HIV-negative patients with PCP, there is no evidence that adjunctive steroid therapy is beneficial (39,40). A review of 31 non-HIV-positive patients with confirmed PCP and hypoxemia found that those who received a higher dose of steroids had a shorter duration of mechanical ventilation and oxygen use (39). However, another similar study was unable to show an improvement in survival (40). A further study (42), which concluded that high-dose steroid therapy was associated with increased mortality in HIV-negative patients with PCP via a mechanism independent from an increased risk of infection, also supported this view. All of the patients analyzed in the present study were HIV-negative, and in some of them, PCP was associated with corticosteroid use; 72.22% of the patients received adjunctive corticosteroid therapy. Among them, nine (69.23%) patients eventually experienced treatment failure, which might also be associated with their disease severity. Currently, whether to use corticosteroids or not and their appropriate doses requires serious consideration in severe cases of PCP in HIV-negative patients (41).

No common DHPS gene mutations of *P. jirovecii* were detected in the HIV-negative PCP patients in the present study. However, other mutations were present, the significance of which remains to be further identified. The elevation of neutrophil counts in BALF and reduction of lymphocyte counts in peripheral blood may be associated with poor outcome. The efficacy of adjunctive steroid therapy in HIV-negative PCP patients requires further investigation.

## Figures and Tables

**Table I:** Patient demographics at diagnosis of *Pneumocystis* pneumonia in 18 patients.

Characteristic	Number
Age, years (range)	51 (23–77)
Male gender (%)	12 (67)
Underlying disease (%)	
Inflammatory disease	
Pemphigus erythematosus	1 (6)
ANCA-associated systemic vasculitis	3 (17)
Connective tissue disease	3 (17)
Chronic glomerulonephritis	1 (6)
Malignancy	
Solid tumor	3 (17)
Hematologic malignancy	2 (11)
Organ transplantation	
Bone marrow	1 (6)
Kidney	3 (17)
Other	2 (11)

Some patients had two or more underlying conditions. ANCA, ant-neutrophil cytoplasmic antibody.

**Table II:** Laboratory parameters in patients with *Pneumocystis* pneumonia.

Parameter	Value
White blood cell count, ×10^9^/l (range)	8.22±5.01 (1.47–20.70)
>10×10^9^/l (%)	6/18 (33.33)
<4×10^9^/l (%)	2/18 (11.11)
Neutrophil count, ×10^9^/l (range)	6.99±4.00 (0.59–14.67)
Neutrophil percentage (range)	83±14.75 (37.30–97.30)
>70% (%)	15/18 (83.30)
CD4^+^ lymphocyte count (range)	95 (41.50–150.69)^a^
<200/μl (%)	9/10 (90.00)
C-reactive protein, mg/l (range)	55.80 (20.30–120.50)^a^
>8 mg/l (lower limit of normal, LLN) (%)	12/13 (92.31)
Lactate dehydrogenase, IU/l (range)	427.42±267.84 (51–1137)^a^
>240 IU/l (LLN) (%)	9/12 (75.00)
β-D-glucan, pg/ml (%)	
β-D-glucan >50 pg/ml	6/14 (42.86)
10 pg/ml< β-D-glucan <50 pg/ml	4/14 (28.57)
Bronchoalveolar lavage fluid differentials	
Lymphocytes, % (range)	24.72±16.43 (1–56)
Lymphocyte elevation predominant (%)	3/18 (16.70)
Neutrophils, % (range)	35.56±26.09 (2–86)
Neutrophil elevation predominant (%)	6/18 (33.33)

a Incomplete data.

Data are presented as mean ± standard deviation, or median and interquartile range.

**Table III:** Prognostic factors.

Variable	Non-survivors	Survivors	P-value
Age, years	52.89±10.89	49.22±5.18	0.479
Male, no. (%)	5 (55.6)	7 (77.8)	0.331
Peripheral neutrophils, %	91.24±5.18	75.44±17.18	0.003^a^
Peripheral lymphocytes, % (range)	4.2 (2.4–5.85)	10.1 (5.65–23.4)	0.019^a^
Lactate dehydrogenase, IU/l	416±84.29	435±355.81	0.908
CD4^+^ lymphocyte, n/μl (range)	93 (54–209)	97 (39–165.80)	0.703
C-reactive protein, mg/l (range)	69.1 (13.5–140)	55.15 (26.1–89.55)	0.454
β-D-glucan >50 pg/ml (%)	4/6 (66.6)	2/8 (25.0)	0.170
β-D-glucan >10 pg/ml (%)	5/6 (83.3)	5/8 (62.5)	0.580
PaO_2_/FiO_2_	164.79±78.44	245.73±114.32	0.106
Lymphocytes in BALF, %	18.44±18.60	31.00±11.80	0.107
Neutrophils in BALF, %	49.78±27.67	21.33±15.03	0.047^a^

a Multivariate analysis cannot be achieved due to small sample size.

BALF, bronchoalveolar lavage fluid; PaO_2_, partial pressure of oxygen in arterial blood; FiO_2_, fraction of inspired oxygen.

**Table IV:** *Pneumocystis* pneumonia treatment and outcome.

Treatment	Total	Non-survivors	Survivors	Mortality (%)
TMP-SMZ	16	8	8	50
TMP-SMZ + caspofungin	8	6	2	75
Primaquine + clindamycin	3	2	1	66.67
Adjunctive corticosteroids	13	9	4	69.23

TPM, trimethoprim; SMZ, sulfamethoxazole.
